# Using the Association of American Medical Colleges Standardized Video Interview in a Holistic Residency Application Review

**DOI:** 10.7759/cureus.1913

**Published:** 2017-12-06

**Authors:** Andrew King, Chad Mayer, Andrew Starnes, Kelly Barringer, Lancelot Beier, Harsh Sule

**Affiliations:** 1 Emergency Medicine, The Ohio State University Wexner Medical Center; 2 Emergency Medicine, The University of Oklahoma - Tulsa; 3 Emergency Medicine, Regions Hospital; 4 Emergency Medicine, Virginia Commonwealth University; 5 Emergency Medicine, Rutgers New Jersey Medical School

**Keywords:** standardized video interview, american association of medical colleges, holistic application review, holistic application review

## Abstract

Each year, residency programs work diligently to identify the best applicants for their respective programs, given the increasing volume of applications. Interview offers are often based on a mix of subjective and objective measures, with different programs relying more or less on each. A holistic application review involves a flexible and individualized way of assessing an applicant’s capabilities through a balanced consideration of experiences, attributes, and academic metrics. When considered collectively, these attributes may define how an individual may perform as a physician. One particular tool developed by the American Association of Medical Colleges (AAMC), the Standardized Video Interview (SVI), provides an objective measure of an applicant's professional behavior and interpersonal communication skills. The SVI may provide applicants with a chance to showcase the intangibles about themselves that are neither entered on their application nor reflected by their standardized examination scores.

## Introduction

Each year, residency programs work diligently to identify the best applicants for their respective programs, given the increasing volume of applications. Interview offers are often based on a mix of subjective and objective measures, with different programs relying more or less on each. Objective measures, which are more available, such as class rank, away rotation grades, or the United States Medical Licensing Exams (USMLE) 1 and 2 scores, may predict success in residency, but the literature is inconsistent [1-2]. Success on standardized examinations may predict success on future board exams, but this is not the same as performance while in training [2]. Examples of subjective measures include the Standardized Letter of Evaluation (SLOE), used in Emergency Medicine (EM). The SLOE hopefully helps programs develop a more accurate understanding of an applicant’s performance in medical school when compared to free-form letters, such as the Medical Student Performance Evaluation (MSPE) [3]. The SLOE is meant to be standardized and concise while providing evaluative data such as commitment to EM, ability to work with a team, work ethic, ability to develop a cohesive treatment plan, ability to communicate with patients, and the amount of guidance an applicant will require when compared to peers. Unfortunately, the SLOE is not without its own limitations [2]. As in the MSPE, letter writers tend to provide more positive reviews regarding medical students affiliated with their institutions because negative SLOEs may inhibit an applicant’s chance of matching in a residency program, which ultimately reflects poorly on the applicant’s medical school. The holistic application review of an applicant, with a balance of subjective and objective evaluations, may be the ideal approach to choose residents; however, defining and understanding the importance of this method can be challenging.

## Technical report

A holistic application review involves a flexible and individualized way of assessing an applicant’s capabilities through a balanced consideration of experiences, attributes, and academic metrics. When considered collectively, these attributes may define how an individual might perform as a physician. In a holistic application review process, selection criteria are broad-based, clearly linked to departmental and program goals, and promote diversity as a critical element to institutional success. A balanced consideration of experiences, attributes, and academic metrics is used to review applicants to ensure a diverse residency class. Race and ethnicity may be considered factors when making decisions only when such consideration is narrowly tailored to achieve mission-related educational interests and goals associated with diversity, and when considered as part of a broader mix of factors, which may include personal attributes, experiential factors, and demographics [4]. Diversity is a relatively new target for how to structure a program and represents one with which academic medical centers have historically struggled, particularly in leadership positions [3]. New strategies are emerging to help programs promote diversity at all levels within academic medical centers [5-6]. At the residency program level, a specific goal of a holistic review is to identify candidates who would perform as good as or better than those considered through purely algorithmic selection, while promoting diversity through varied experiences [7].

The medical education literature has attempted to identify predictive factors for future success in residency training. While some education research has pointed to medical school attended or standardized examination scores as predictors of future success in residency, there is agreement that no single or a few definitive criteria can make this prediction confidently [8-10]. As the number of applicants continues to increase, it has become clearer that programs need ways to evaluate applicants interpersonally and professionally. Measuring both the numbers and the interpersonal characteristics is the goal of a holistic application review. Currently, as part of the holistic application review process, a great tool to evaluate an applicant’s professionalism and communication skills is not available, aside from a clinical rotation or a formal interview. Inevitably, many applicants each cycle do not have the opportunity to demonstrate their interpersonal skills or professionalism to a program by one of these methods, given the large number of applicants for limited external clinical rotations. The Standardized Video Interview (SVI) developed by the American Association of Medical Colleges (AAMC) can provide data that contributes to the holistic review of an applicant, especially pertaining to their professionalism and communication skills. The SVI was created by the AAMC out of a desire by program directors to have a way to measure this facet of an applicant prior to interview selection and in a standardized manner, especially given the inability to identify this information via standard application materials.

The SVI is being piloted with emergency medicine (EM) residency applicants during the 2018 application cycle. It is composed of six questions designed to assess an applicant’s knowledge of professional behaviors as well as interpersonal communication skills. SVI questions are randomly generated from a collection of validated questions owned by the AAMC. The interview videos are scored by third-party raters that have undergone extensive inter-rater reliability testing. A composite score is included within each emergency medicine residency applicant’s Electronic Residency Application Service (ERAS) file. Videos are made available to residency programs for direct review. For the ERAS 2018 cycle, AAMC has strongly advised that all applicants to ACGME-accredited EM programs complete the SVI. While those that do not complete will still be eligible to apply, residency programs will be informed that the SVI was not completed.

## Discussion

The SVI may provide applicants with a chance to showcase the intangibles about themselves that are neither entered on their application nor reflected by their standardized examination scores. Within their education materials describing the SVI, the AAMC states that the SVI has been shown, based on preliminary data, to not correlate with USMLE Step 1 scores. Consequently, the SVI is hopefully accomplishing its overarching goal: measure an aspect of a residency applicant’s worthiness that is not captured by their performance on a standardized examination. Each program will have the opportunity to view an applicant’s SVI to develop a better understanding of the candidate’s fit within the residency program. Scoring highly on the SVI could be an alternative way to convince program leaders to view one’s SVI and possibly extend an interview offer. Conversely, a low score on the SVI for traditionally stellar applicants could result in fewer interview offers extended, improving their perceived monopoly on the desirable interview slots. An SVI score will be provided as part of each emergency medicine residency application. Residency programs will have the opportunity to view the videos; however, this practice will likely be reserved for a few, specific applicants given the significant time burden a holistic application review entails.

The interpretation and incorporation of the SVI in a holistic application review will vary amongst residency programs. Ideally, application reviewers will utilize best practices defined in the literature that apply specifically to candidate selection for residency programs [3]. These include promoting diversity, using multiple, different objective assessments, and considering the unique experiences and attributes of applicants. The SVI can function as a surrogate interview that program leadership can choose to view. By considering the SVI when performing a holistic application review, a program should receive a more complete and personal understanding of each individual. Similarly, the SVI will provide programs with a tool to assess interpersonal communication and professionalism prior to a formal interview. This tool can provide a significant impact by ensuring that each applicant gets an interview, albeit on standardized questions. Residency program leadership should utilize this unique data in order to ensure an entirely holistic understanding of an applicant.

## Conclusions

The AAMC’s plan to formally evaluate the success of the SVI currently remains unclear. Whether it is post-cycle surveys, or a more rigorous statistical analysis of the performance of interns who first match using the SVI, it is clear that we are a long way from understanding whether this tool will be important to future residency cycles. If successful in some form, its use will likely be expanded to other specialties beyond emergency medicine. What remains clear is the increased need to evaluate residency applicants holistically, rather than placing excessive weight on examination scores and medical schools attended. A robust plan to measure outcomes and assess the impact of the SVI, especially in applicants who may have been otherwise passed over, is needed to formally assess its value. 
